# Digital Health Apps in the Context of Dementia: Questionnaire Study to Assess the Likelihood of Use Among Physicians

**DOI:** 10.2196/35961

**Published:** 2022-06-22

**Authors:** Markus Schinle, Christina Erler, Mayumi Kaliciak, Christopher Milde, Simon Stock, Marius Gerdes, Wilhelm Stork

**Affiliations:** 1 Medical Information Technology Embedded Systems and Sensors Engineering FZI Research Center for Information Technology Karlsruhe Germany; 2 Biological Psychology Department of Psychology University of Koblenz and Landau Landau Germany; 3 Institut fuer Technik der Informationsverarbeitung Department of Electrical Engineering & Information Technology KIT Karlsruhe Institute of Technology Karlsruhe Germany

**Keywords:** digital health applications, likelihood of use, usability, adherence, dementia, screening, treatment, physician, eHealth, questionnaire, mobile phone

## Abstract

**Background:**

Age-related diseases such as dementia are playing an increasingly important role in global population development. Thus, prevention, diagnostics, and interventions require more accessibility, which can be realized through digital health apps. With the *app on prescription*, Germany made history by being the first country worldwide to offer physicians the possibility to prescribe and reimburse digital health apps as of the end of the year 2020.

**Objective:**

Considering the lack of knowledge about correlations with the likelihood of use among physicians, this study aimed to address the question of what makes the use of a digital health app by physicians more likely.

**Methods:**

We developed and validated a novel measurement tool—the Digital Health Compliance Questionnaire (DHCQ)—in an interdisciplinary collaboration of experts to assess the role of proposed factors in the likelihood of using a health app. Therefore, a web-based survey was conducted to evaluate the likelihood of using a digital app called DemPredict to screen for Alzheimer dementia. Within this survey, 5 latent dimensions (acceptance, attitude toward technology, technology experience, payment for time of use, and effort of collection), the dependent variable *likelihood of use*, and answers to exploratory questions were recorded and tested within directed correlations. Following a non–probability-sampling strategy, the study was completed by 331 physicians from Germany in the German language, of whom 301 (90.9%) fulfilled the study criteria (eg, being in regular contact with patients with dementia). These data were analyzed using a range of statistical methods to validate the dimensions of the DHCQ.

**Results:**

The DHCQ revealed good test theoretical measures—it showed excellent fit indexes (Tucker-Lewis index=0.98; comparative fit index=0.982; standardized root mean square residual=0.073; root mean square error of approximation=0.037), good internal consistency (Cronbach *α*=.83), and signs of moderate to large correlations between the DHCQ dimensions and the dependent variable. The correlations between the variables *acceptance*, *attitude toward technology*, *technology experience*, and *payment for the time of use* and the dependent variable *likelihood of use* ranged from 0.29 to 0.79, and the correlation between *effort of the collection* and *likelihood of use* was −0.80. In addition, we found high levels of skepticism regarding data protection, and the age of the participants was found to be negatively related to their technical experience and attitude toward technology.

**Conclusions:**

In the context of the results, increased communication between the medical and technology sectors and significantly more awareness raising are recommended to make the use of digital health apps more attractive to physicians as they can be adjusted to their everyday needs. Further research could explore the connection between areas such as adherence on the patient side and its impact on the likelihood of use by physicians.

## Introduction

### Background

The populations in Germany and the United States are characterized by a growing proportion of people aged >60 years [1,2]. As an age-related disease, Alzheimer dementia is of particular importance in our society [3,4] as approximately 5% of people aged >65 years already experience severe dementia, and another 10% experience mild to moderate dementia [5]. Owing to this demographic development, the number of patients with dementia is increasing [6,7]. As early detection of dementia offers the patient the opportunity to manage daily issues such as finances and insurance by themselves [8], it has led to the rising importance of dementia detection, especially in the early stages [9,10].

Dementia is a disease with various causes that entails an above-average loss of intellectual skills. The main group of people affected is in an advanced age of >65 years [5].

The course of dementia is usually chronic or progressive, affecting the higher cortical functions (eg, memory, orientation, cognition, learning, language, and judgment), motivation, social behavior, and emotional control of the person with the disease [11].

Approximately 60% of patients with dementia experience the so-called Alzheimer disease, which usually begins insidiously and leads to death after approximately 5 to 10 years [5].

To support the early detection of dementia, it is possible to use mobile solutions such as apps on smartphones or tablets. In this way, the financial burden on the health system can be reduced [12,13] as there is a time saving of up to 30% in comparison with traditional solutions [14]. There are further studies worldwide that also report time savings by using digital solutions (eg, in Austria [15], Germany [14,16-18], and the United States [12,13,19]), including the special case of dementia screening [16].

Owing to the ease of accessibility [14], many physicians and patients like to use smartphones for remote diagnosis [20]. Furthermore, physicians describe apps for diagnosis as the most useful implementation in the eHealth sector [21].

In addition, an interdisciplinary exchange is facilitated in the use of technological tools [22], which is good for research and provides a more accurate picture of diseases such as dementia because of larger data sets available and the possibility of monitoring patients longitudinally with a high temporal resolution [14]. Therefore, universities and the health care systems of every country should have a great interest in developing and promoting the use of technology [23].

Hereby, an improvement in treatment can be established [24,25], and medical apps can be considered very useful tools in prevention and therapy [15] as they are considered not only for use in diagnosis but also for remote monitoring of patients [21]. An example is monitoring patients with Alzheimer disease using the Android app iWander [4] to analyze their location using GPS to make life easier and minimize financial burden [20].

Apps such as these make it easier to care for patients with chronic diseases [21], and diagnosis apps permit early intervention, for example, through cognitive training for Alzheimer disease, which has been shown to have a positive impact on disease progression [26].

### Prior Work

In Germany, approximately 1 in 5 physicians stated that they use technical aids in their contact with patients (eg, to support patient information or diagnostics), making this a frequently used device [27].

To analyze what exactly influences the use of technical aids, there are some studies in the United States in which it was found that a positive attitude toward smartphones or toward one’s own ability to use them has a positive influence on the frequency of use [28,29].

Other groups of researchers have found that even observability, compatibility, job relevance, personal experience, and the internal and external environment influenced the attitude toward using a smartphone [30] and that the perceived usefulness also has an impact [29].

There are 3 groups of physicians: one-third are neutral, one-third welcome technical progress, and the other third is skeptical [31], especially concerning data security [18,22,24,25,27] and confidentiality [32,33], legal ambiguities, or a risk of abuse [18].

However, according to another study, there seem to be sophisticated approaches that increase transparency in apps for the user (eg, the physician) [13]. There are also strict rules for apps in Germany to be covered by health insurance via prescription as this requires a certification as a medical device [34] and also that safety can be ensured [35]. In the United States, it also depends on what the app is to be used for and which rules apply to it. For example, medical apps need approval from the Food and Drug Administration or a certification from private sources; however, these certifications are expensive [13].

Therefore, many app developers circumvent these often long-lasting certification processes by not considering the billing of health insurance as an option or by declaring their app only as an accompaniment but not a diagnostic instrument. The latter explains why there are some apps on the market for digital dementia screening that often do not fulfill medical criteria, such as those provided by the Diagnostic and Statistical Manual of Mental Disorders, Fifth Edition [36], for testing complex attention, executive functions, learning, and memory or language [37,38], which explains the physicians’ demand for a binding test seal [39].

Many app developers also disclose little information in the apps [40]. An attempt to establish a reliable seal for consumers in Germany is the *HealthOn-Apps Ehrenkodex* (code of ethics), which is intended to provide guidance by checking the app for a data protection notice, author information, source information, freedom from advertising, financing information, contact data, and imprint [40].

Another point to consider when using apps in medicine is the age of the physician and the patient. Older patients usually make more frequent visits to physicians because of age-related conditions but are the least likely group to use smartphones [13]. For example, in Austria, approximately 64% of younger individuals (aged <44 years) and only approximately 39% of older individuals (aged >44 years) use a smartphone [15]. Unfortunately, however, age and age-related limitations are often not considered by developers, which leads to avoidable difficulties in use [27,41].

This is happening even though it has long been known that differences in attitudes toward technology can be attributed to the age and educational level of the participants (physicians and patients) [27]. For example, a Swiss study identified a group of younger participants among physicians with a positive attitude [42], and a German study found that younger participants rated the opportunities for digitization significantly higher (aged 20-29 years: 93% compared with aged >70 years: 44%) [18].

### Goal of This Work

There is limited research worldwide on the acceptance of mobile health technologies among physicians [43], and the mass of offerings (apps) is difficult to sort through [44]. In addition, most research is limited to the patient side [45], which is why more attention should be paid to the needs of physicians in the development that is already taking place [13,34]. To this end, a collaboration between behavioral researchers and developers is of great importance and could lead to better results concerning the health care system [13].

When it comes to the question of how exactly an app must be designed so that physicians are willing and motivated, the factors that correlate with the likelihood of use should be examined more closely. A well-designed app could support the diagnosis, disease monitoring, and treatment of patients with chronic diseases, especially in low-income countries [13]. Moreover, medicine in general could achieve more efficient information exchange and collaboration [14,22] and, thus, better care [24,25].

The test theoretical evaluation of the Digital Health Compliance Questionnaire (DHCQ) using confirmatory factor analysis (CFA) is to be defined as an initial goal whereby the latent factors, as well as the items that make up these factors, were selected by an interdisciplinary expert committee. A further objective was to answer the guiding research questions of (1) which *physician needs* are related to a high likelihood of using health apps and (2) which *physician characteristics* (skills and attitude toward technology) are correlated with an increased likelihood of using health apps. In addition, it is an example of interdisciplinary collaboration, thus uncovering skepticism and using it as a basis for better education to be able to accomplish (as recommended [10]) more early Alzheimer diagnoses and, thus, increase the rate of affected individuals who are treated (currently at only approximately 50% [14]).

### Hypotheses

It is to be examined which dimensions, in general, are related to physicians’ likelihood of use. On the basis of the aforementioned indications from the literature and an expert panel consisting of psychologists, geriatricians, pedagogues, and engineers with years of experience in the field of eHealth, the factors to be investigated were defined. For this purpose, *attitude toward technology*, *technology experience*, *payment for the time of use*, and the *effort of the collection* will be considered as independent variables, and *likelihood of use* will be considered as a dependent variable. To be able to apply the data to an example, special items were developed that capture the independent variable *acceptance of the app*. For this purpose, the functionality of the digital dementia screening app DemPredict [16,46] was briefly explained in a free field of this study, and some questions on its acceptance were formulated.

From these 6 dimensions, five hypotheses can be derived: (1) the likelihood of using the app is positively related to its *acceptance* (hypothesis 1), (2) the likelihood of using the app is positively related to the *attitude toward technology* (hypothesis 2), (3) the likelihood of using the app is positively related to *technology experience* (hypothesis 3), (4) the likelihood of using the app is positively related to the *payment* for the time of use (hypothesis 4), and (5) the likelihood of using a survey method is negatively related to the *effort of collection* (hypothesis 5).

In addition to the main hypotheses, secondary research questions were posed, which will be discussed in more detail in the Results and Discussion sections: (1) Does *age of the test person* correlate with *technology experience* as well as *attitude toward technology*? (2) Does it matter whether the app is declared as a medical device? (3) What role do concerns about data protection play? (4) Do physicians think that the time required for a dementia screening via app is higher for older people? (5) Is digital support for early monitoring of disease progression desired? (6) Can physicians imagine having a screening carried out under the supervision of a physician’s assistant or would they even trust a result brought from home or a test carried out alone in the waiting room?

## Methods

### Research Design

The data collection was implemented as a web-based survey using the tool SoSci Survey (version 3.2.21; SoSci Survey GmbH) and was conducted in February and March 2021. After adjustment, 301 participants remained to be included in the evaluation (*Sampling Design and Recruitment*). Statistical analysis was conducted using the statistical programming language R (version 4.0.4; R Foundation for Statistical Computing) as implemented in the R Studio environment. The CFA was used with the packages lavaan [47] and semPlot [48] using the commands *sem* and *semPaths*. Correlational hypotheses were tested using the command *cor.test* and illustrated using the package ggplot2 [49].

### Sampling Design and Recruitment

The required sample size was estimated using G*Power (version 3.1.9.7; University of Düsseldorf) assuming an *α* of .05 and a power of 1 – *β* of .80. Despite intensive research, we could not find any comparable studies that would have made a priori sample size planning possible. The effect size was estimated by the consortium to be small (*r*=0.1 according to Cohen [50]) to obtain a feeling for the implementability of the study. This resulted in a sample size of 67 participants. Compensating for an attrition rate of 15%, we had to acquire ≥78 participants.

**Figure 1:**
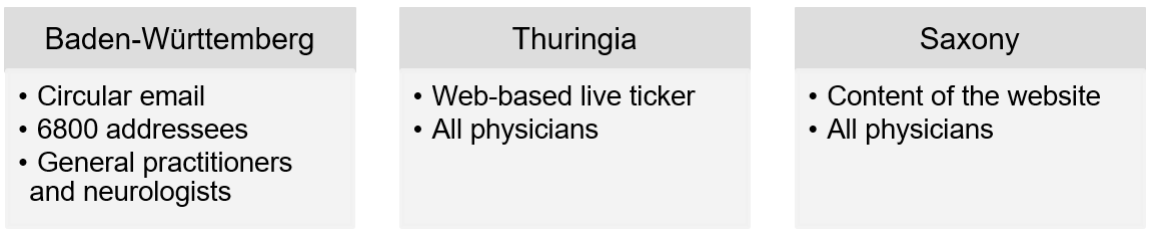
Recruiting channels.

To recruit physicians, we followed a non–probability-sampling strategy and contacted all of the 16 regional Associations of Statutory Health Insurance Physicians (Kassenärztliche Vereinigungen) in Germany, the Association of General Practitioners (Hausärzteverband), the German Medical Association (Bundesärztekammer), and the German Society for General Practice and Family Medicine e.V. On the basis of these initial contact requests, a successful contact was established with the Associations of Statutory Health Insurance Physicians of the states of Baden-Württemberg, Thuringia, and Saxony. The physicians were recruited through the channels shown in Figure 1.

### Study Sample

**Figure 2:**
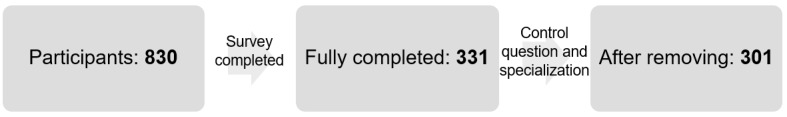
Study sample.

A total of 830 physicians participated in the study (Figure 2). Physicians who did not complete the survey were excluded from the study (fully completed: 331/830, 39.9% of the participants). In addition, there were 2.3% (19/830) of participants who only insufficiently or not at all completed the control question *To ensure the evaluability of the study, please briefly describe in your own words what the DemPredict application is supposed to do* (test participants 55, 147, 218, 224, 279, 360, 369, 417, 419, 452, 468, 480, 541, 593, 601, 647, 656, 709, and 731).

Furthermore, 11 physicians from nonrelevant specialties (definitely not in contact with patients with dementia or involved in dementia diagnosis) worked on the study (test participant 61, research associate; test participant 330, cardiology; test participant 574, orthopedics; test participant 587, dermatology; test participant 641, surgery; test participant 649, oncology; test participant 771, pediatrics; test participant 786, urology; test participant 797, gynecology; test participant 837, trauma surgery or orthopedics; and test participant 870, anesthesiology). They were also subsequently removed from the data set.

### Ethics Approval

The study was approved with ethical approval number LEK-319 (application date April 02, 2021) by the local ethics committee of the University of Koblenz-Landau, Germany. The study is replicable because of the transparent design of the questionnaire. All participants were informed of the voluntary nature of their participation, their anonymity, and the possibility of asking questions. They agreed to the privacy policy by marking a checkbox in the web-based questionnaire.

### Data Collection: Factors Determining Likelihood of App Use

Data collection was performed with a designed questionnaire called the DHCQ (Multimedia Appendix 1 [26]), which was partially based on items from other studies [51-53]. It is divided into 7 sections. In addition to demographic data, the survey asked about technology experience and attitudes toward technology. The questionnaire encompassed open- and close-ended or multiple-choice items. Information on the app DemPredict was followed by questions about attitudes toward this app and the requirements that such an app must fulfill to be used successfully in standard care.

The 2 dimensions *attitude toward technology* and *technology experience* are common dimensions in the literature [51-53], to which only new items were added. To define the new dimensions *acceptance*, *survey effort*, and *payment*, an interdisciplinary expert committee of psychologists, geriatricians, pedagogues, and engineers was formed, which discussed the items for these new dimensions.

To verify the proper understanding of the survey items and to reduce additional errors such as spelling or formatting errors, several pilot data sets were acquired within the research group by 3 members of the medical staff and 3 engineers beforehand.

### Statistical Analysis

#### Overview

The 5 main hypotheses were all directly correlational. Thus, 1-sided Pearson correlations with a significance threshold of *P*=.05 were used to assess statistical significance. The Pearson correlation coefficient (*r*) was used as an effect size measure.

#### Item Analysis

Descriptive statistics were used to assess the suitability of the items and scales for the subsequent CFA and correlational analysis. Therefore, we computed measures of location (arithmetic mean and median), dispersion (SD), and shape (skewness and kurtosis; Tables S1 and S2 in Multimedia Appendix 2). To assess the normality of the items, a skew value of >2 or kurtosis of >7 was considered nonnormal [54]. The level of discrimination should be >0.3 to be *good* and >0.39 to be *excellent* [55]. Difficulty in this questionnaire was not similar to measuring the difficulty of solving an item but can be defined as *endorseability* [56] because a Likert scale was used. A difficulty range of 30 to 60 was *good*, a lower range was *difficult*, and a higher range was *easy* [57].

To interpret the *P* values from the Pearson correlations for the main hypothesis, the assumption of bivariate normality must be fulfilled. To test this assumption, the Shapiro-Wilk test was used. We used bias-corrected and accelerated bootstrapped Pearson correlations to obtain robust correlation statistics (1000 iterations).

#### CFA Component

To test the validity of the measurement model and to illustrate correlations, we decided to use a CFA to support the previous theoretical considerations.

**Figure 3:**
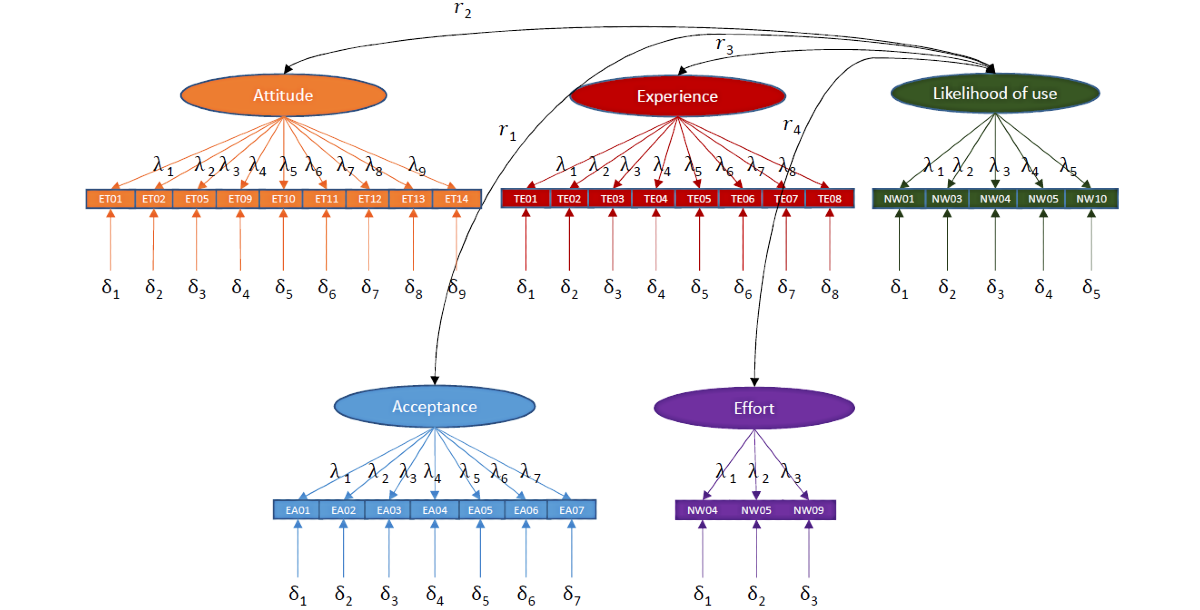
Measurement model. r: correlations; λ: loads; δ: error variances; ET: Attitude toward Technology; TE: Technical Experience; NW: Probability of Use; EA: Attitude toward App Acceptance.

The graphical representation of the parameters is shown in Figure 3.

From this, we can define formulas, of which one is shown as follows using 1 item as an example: *EA*01 = *λ*1 × *acceptance* + 0 × *attitude* + 0 × *experience* + 0 × *effort* + 0 × *likelihood of use* + *δ*1.

The CFA is overidentified as, with 68 unknown variables—loads (*λ*; 32) + error variances (*δ*; 32) + correlations (*r*; 4)—a total of 528 known variables can be identified (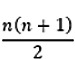 with n=32). Thus, parameter estimation and goodness-of-fit testing were possible. To check whether the model-theoretical covariance matrix differed significantly from the observed matrix, parameter estimation was performed [58]. Owing to the very coarse scale (values from 1 to 5), the skewness of the items and scales [59] (*Item and Scale Analysis*), and the ordinal scaling of the items [60], parameter estimation should not be performed using the maximum likelihood method for these data. Nevertheless, to be able to test the a priori assumption regarding the latent constructs (measurement model), we decided to perform parameter estimation using the diagonally weighted least squares (DWLS) estimator, which is more robust for nonnormally distributed and ordinal data [60], to be able to evaluate the model fit in the next step.

To describe the fit of a model, fit indexes such as the standardized root mean square residual, the root mean square error of approximation, the comparative fit index (CFI), and the Tucker-Lewis index (TLI) were used. For the badness-of-fit measures (standardized root mean square residual and root mean square error of approximation), lower values (approximating 0) indicated a high model performance and, for the goodness-of-fit measures (CFI and TLI), higher values (approximating 1) indicated a high model performance [61]. As the model chi-square is not very reliable for large samples, especially with skewed item distributions, it will not be considered as a model performance measure in our sample because the dependence of the probability of error *β* on the sample size [58] quickly results in a significant value for 301 participants.

Everything needed to determine validity is found in the output of the CFA [62,63]. Convergent validity can be described by good factor loadings, which do not cross-load on nonrelevant constructs [64]. Both convergent and discriminant validity are represented in good model fit indexes (in this study, CFI and TLI) [61]. Internal consistency was assessed using the Cronbach *α* [64], with *α*>.70 being recommended and *α*>.90 considered as redundant [64-66].

## Results

### Overview

The results of this study are presented in the following sections. First, the distribution of the demographic data in the sample is shown, followed by a description of the item and scale scores and the results of the CFA. After that, we examine the reliability and validity of this study and, finally, explain the results of the hypotheses and exploration.

### Sample Statistics

The age of the participants was approximately recorded by age groups to simplify the evaluation. This ranged over 4 groups specified in years (<30 years, 30-45 years, 45-60 years, and >60 years), which is why it is not useful to specify the mean and SD.

When specifying the gender, there were 3 selection options in the questionnaire (male, female, and diverse), and it can be said that the sample was balanced regarding gender types *Male* and *Female*. In this study, the specialty of the attending physician played a role as domain knowledge about dementia should be present, which is why it was included in the survey. All sociodemographic data are shown in Table S3 in Multimedia Appendix 2.

### Item and Scale Analysis

**Figure 4:**
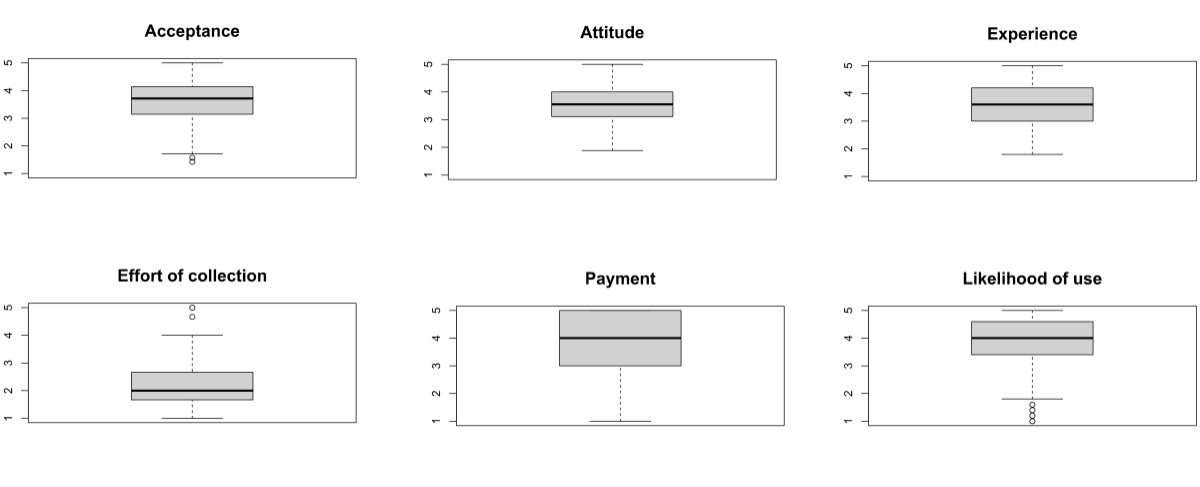
Box plots of dimensions from does not apply (1) to applies (5), inverted for effort.

All measures of central tendency and dispersion can be found in tables with item and scale values in Multimedia Appendix 2. It is noticeable that the items and scales partly show skewness. Moreover, as the data are ordinally scaled and the scale is only 5 levels, the methodology of the CFA was adapted (DWLS instead of maximum likelihood as parameter estimation). The difficulty range in 12 items was between 30 and 60 and, in the other items, between 60 and 82. Regarding the fact that items such as *Electronic devices make my everyday life easier* or *A wisely designed application can support an anamnesis just as well as a paper test* clearly are easier to respond to, these were acceptable values to continue the evaluation [56,57]. The level of discrimination in all items except the item that represents the effort for testing with tools such as DemPredict under the supervision of an assistant (item NW09.m: 0.08) was between 0.27 and 0.79, which are good to excellent values (Table S1 in Multimedia Appendix 2). In addition, the scales were tested for their normal distribution using the Shapiro-Wilk test to check the characteristic values. It can be seen that they are not normally distributed except for the dimension *attitude toward technology* (Table S2 in Multimedia Appendix 2). However, owing to the sample size of 301 participants, the data could be evaluated and interpreted [54,58]. As shown in Figure 4, the dimensions *acceptance*, *attitude*, *experience*, *effort of collection*, and *likelihood of use* were all checked for outlier values. No abnormalities were found either.

### CFA Component

Now that the measurement model has already been specified, the model fit is examined in more detail and the results are presented. After specifying the measurement model by graphical representation, setting up the equations, and checking the identifiability as well as the parameter estimation using the DWLS estimator, the overall model fit (Table 1) can be judged as *acceptable* [67].

**Figure 5:**
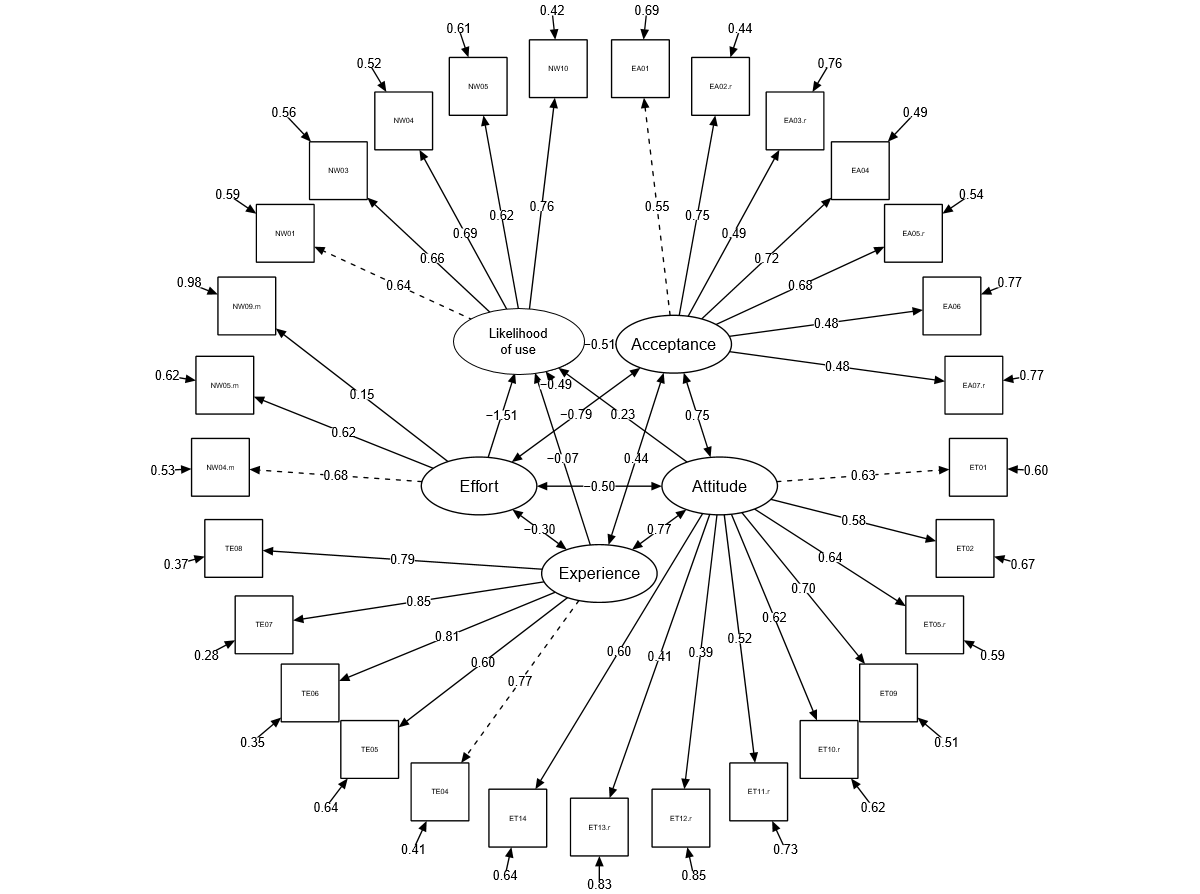
Results of confirmatory factor analysis for structured equation model. ET: Attitude toward Technology; TE: Technical Experience; NW: Probability of Use; EA: Attitude toward App Acceptance.

Thus, the measurement instrument can be classified as functional and used as a basis for further data processing. The presentation of the results of the CFA is shown in Figure 5.

At this point, the correlations of the latent dimensions can be considered, which will be further explored in the follow-up by testing the hypotheses. It can be observed that all items show a satisfactory to very good loading and—matching the acceptable to very good fit indexes—correctly reflect the respective dimensions (Figure 5). A good construct convergent validity was indicated by the high factor loading, which can be classified as *good* (>0.55) [64,68]. Moreover, the goodness-of-fit indexes CFI and TLI (Table 1) indicated a *very good* overall model fit [61] and were also a sign of good discriminant validity [64,69]. As the instrument could generalize well [64] in specialties other than dementia, a good external validity was also expected. It can be used for every health app with only minor adjustments—such as in the dimension *acceptance of the app*, which was adapted to the app DemPredict (*Hypotheses* and Schinle et al [16,46]). The Cronbach *α* indicated a *very good* reliability with a value of .83 (95% CI 0.8-0.86) [65]. The power of the study in the total sample with an *α* error level of .05 and a sample size of 301 participants was 1. As there is a relationship between power, sample size, and postulated effect size and because of the dependence of the probability of the *β* error on sample size [58], the interpretability of the significance of the results was questioned, leading us to take the following further step: using 4 randomly generated subsamples, the power was recalculated. Now, a slightly different picture emerges from the overall sample. With a sample size of 75 and an *α* error level of .05, the average power was 0.966. As, in a Pearson correlation, the correlation coefficient *r* represents the effect size, the effect sizes are also reported and can be used by a replication study or a study in a similar field in a priori sample size planning.

**Table 1:** Model fit indexes.

Indexes	Actual value	Set point
RMSEA^a^	0.037	<0.08
SRMR^b^	0.073	<0.10
CFI^c^	0.982	>0.90
TLI^d^	0.980	>0.95

^a^RMSEA: root mean square error of approximation.

^b^SRMR: standardized root mean square residual.

^c^CFI: comparative fit index.

^d^TLI: Tucker-Lewis index.

### Evaluation Outcomes

#### Main Hypotheses

All hypotheses were tested for robustness (Table 2). For this purpose, previous analyses were performed on the normal distribution (*Item Analysis*). As the sample with n=301 is larger than n=30, it can be assumed that the values are nevertheless robust despite not being normally distributed, and significance tests can be performed [58].

**Figure 6:**
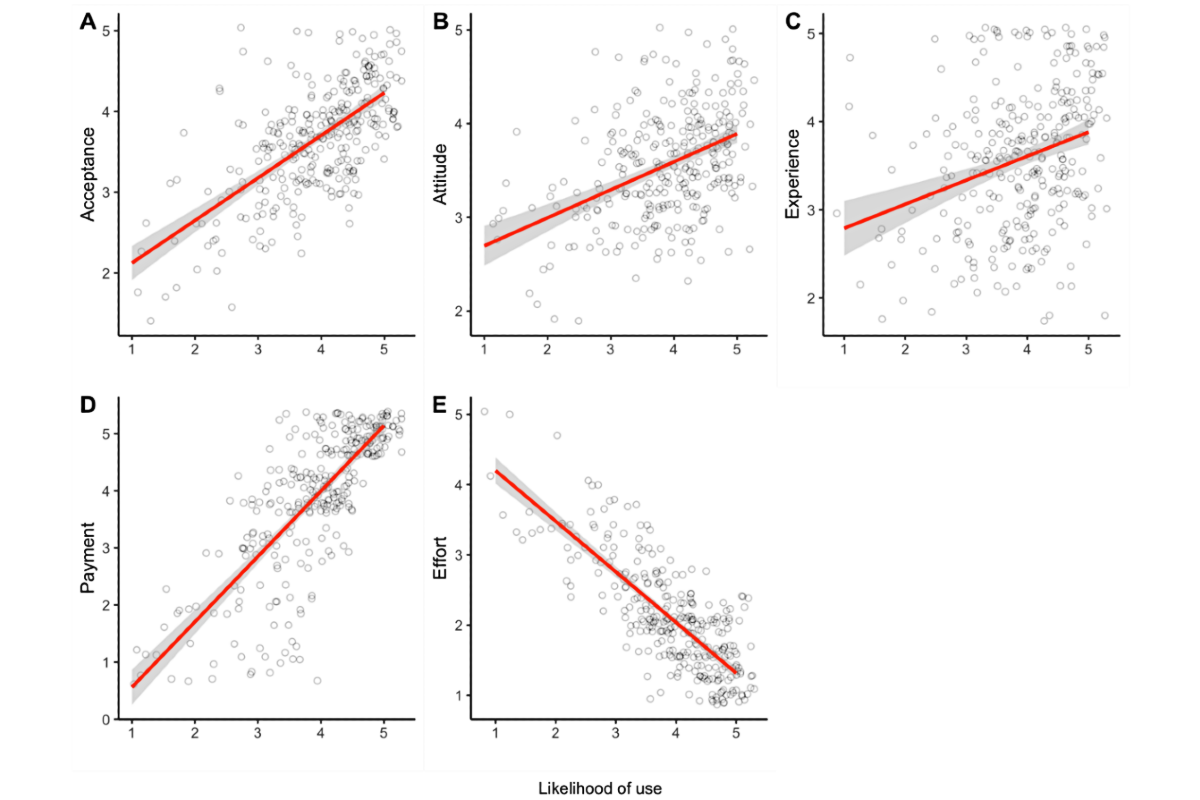
Correlations between items and likelihood of use (A-E).

It can be stated that all 5 hypotheses can be confirmed with good significance values (also considering the sample size) for this purpose compared with the 4 subsamples (see power). All correlations are in a medium- to high-value range and, thus, show clear correlations (Figure 6; Figures S1-S5 in Multimedia Appendix 2).

**Table 2:** Statistical values of the hypotheses.

Factors	1-tailed *t* test (*df*)	*P* value	Correlation (95% CI)	Effect
Acceptance+likelihood of use	14.822 (299)	<.001	0.65 (0.59 to 1.00)	Large
Attitude+likelihood of use	8.356 (299)	<.001	0.44 (0.35 to 1.00)	Medium-large
Experience+likelihood of use	5.228 (299)	<.001	0.29 (0.20 to 1.00)	Medium
Effort+likelihood of use	22.09 (299)	<.001	0.79 (0.75 to 1.00)	Large
Payment+likelihood of use	−22.97 (299)	<.001	−0.80 (−1.00 to –0.76)	Large

#### Secondary Research Questions

##### Correlation of the Age of the Test Person With Technology Experience as Well as Attitude Toward Technology

**Figure 7:**
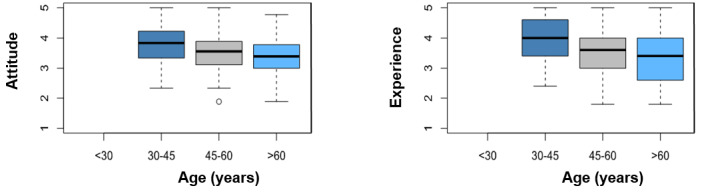
Box plots of attitude and age and of experience and age.

As Figure 7 clearly shows, there is a correlation between technical experience and age, and it can be observed that the median decreases with increasing age. As the graph of attitude toward technology is very similar, it can be assumed that there is a negative correlation between experience and attitude and age (ie, experience decreases and attitude deteriorates with increasing age).

##### Effect of the App Being Declared as a Medical Device

The mean value for item NW07—*If the application is certified as a ‘medical device,’ this increases the likelihood of using it*—was 3.38 (SD 1.29) on a scale of 1 (does not apply) to 5 (applies), which is well above the mean. The modal value of 4 (rather applies) also indicates the clear direction that an app declared as a medical device is better accepted (Figure S6 in Multimedia Appendix 2).

##### Role of Concerns About Data Protection Play

**Figure 8:**
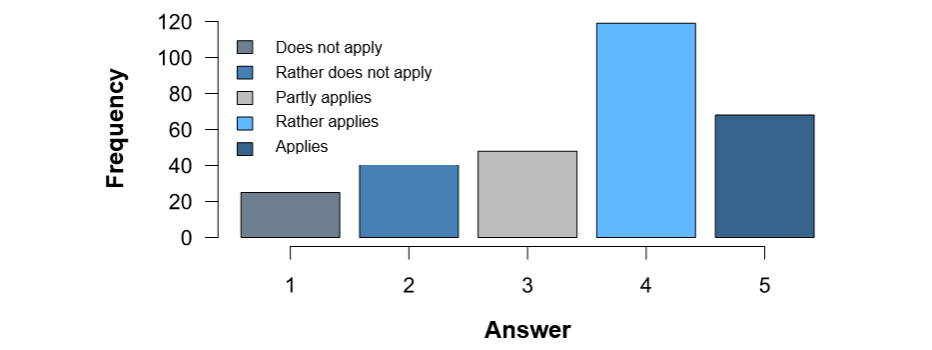
Concerns about data protection.

Item EA03—*I have concerns about problems with data protection*—had a mean value of 3.54 (SD 1.21) on the same scale of 1 to 5, which is also well above the mean. Here, the modal value of 4 also shows a clear tendency toward worries about data protection. As data protection is a very important factor, Figure 8 underlines this statement.

##### Physicians’ Thoughts About Whether the Time Required for a Test via App Is Higher for Older People

Item EA05—*I think it is more time-consuming to use an app with older people than a paper test*—showed a disagreement among physicians, which is also reflected in the mean value (2.97, SD 1.14). Answer option 3 (partly applies) received the most votes, and a symmetrical picture of the remaining answer options was formed whereby critical and uncritical votes balanced each other out (Figure S7 in Multimedia Appendix 2).

##### Need of Digital Support for Early Monitoring of Disease Progression

The question of whether they would like digitally supported monitoring of the course of the disease (item NW12) was answered by 86% (259/301) of the physicians with *yes*. Among the 301 votes, there were 4 (1.3%) abstentions (Figure S8 in Multimedia Appendix 2).

##### Physicians’ Concerns about Having a Screening Carried Out Under the Supervision of a Physician’s Assistant or the Result Brought From Home or a Test Carried Out Alone in the Waiting Room

A total of 48.5% (146/301) of the physicians could not imagine relying on a test result brought in by the patient. Approximately one-third (102/301, 33.9%) of the participants answered the question with *maybe* and partly filled in a free-text field provided with reasons. Processing in the waiting room without supervision also encountered skepticism. The mean value of the question of whether the physicians would allow the test to be performed in the waiting room without supervision was 2.64 (SD 1.22), and the most frequently selected answer was *rather does not apply* (Figure S9 in Multimedia Appendix 2). However, they were very open to the idea of having the test performed under the supervision of a physician’s assistant to save time on the part of the physician. Most respondents decided to select the answer *rather applies* (122/301, 40.5%) or *applies* (59/301, 19.6%). The high mean score (3.52, SD 1.17) also indicates a clear direction in the responses (Figure S10 in Multimedia Appendix 2).

## Discussion

### Principal Findings

As more and more older people live in Germany [1] and the United States [2], age-related diseases such as dementia [3-5,7,9] will continue to increase. Thus, advancing technology to support early diagnosis and low-threshold access to care is becoming more important [8-10,22].

However, for a novel technology to be accepted and more likely to be used, it must be adapted to the different stakeholders. This led us to the guiding research question of what physicians need for the use of an app and what correlations exist on the part of skills and attitudes toward technology.

It turned out that the acceptance of this app was particularly important, with a correlation of 0.65. Attitude toward technology also played a decisive role (*r*=0.43), followed by technology experience (*r*=0.29). However, by far the most important factors were payment for the time of use (*r*=0.79) and the effort of the collection (*r*=−0.80).

Accordingly, the results show high correlations between the latent dimensions and the probability of use by the treatment providers. The expectations of the results were met, the theoretical considerations could be substantiated with partly very high correlations, and the significance was in a very good range even in smaller subsamples. This again allows for a very reliable interpretation and strengthens the importance of the investigated dimensions. From this, conclusions can be drawn for researchers and the need for action can be specified.

### Comparison With Prior Work

To further increase the acceptance of individual apps, efforts need to be more targeted to meet the requirements from physicians’ perspectives. The physicians would like the apps to be easy to use and evaluate and would also like the results to be presented in a comprehensible way (see the free-text fields in Multimedia Appendix 1). The free-to-use app of the World Health Organization—the mhGAP Intervention Guide for mental, neurological, and substance use disorders in nonspecialized health settings from the Mental Health Gap Action Programme—could be a low-threshold entry point [70]. However, there is still a lack of communication between developers and practitioners, which was partly reflected in frustration in this study. Answers in free-text fields, such as “More sales than science, though?” (test participant 227) or “Many people want to make money with physicians...” (test participant 204), show this great skepticism on the part of the medical profession (as well as *Commercialism and Wild West Relationships* [44]) but are counterbalanced by comments such as “I expressly welcome your efforts, as the coming generations will certainly be accustomed to digital formats” (test participant 375), which also fits with the year-on-year comparison in 2016 to 2017, in which the rates for “I think the development is good...” increased significantly from 12.5% to 23.5% [24].

Thus, despite skepticism, it can be assumed that there is an openness among treatment providers to engage with the technology of a specific app, which is evident in comments such as “I would apply it [...] if colleagues report positive experiences.” This also fits with results from previous studies, where 42.6% of respondents voted for “I think the development is good in principle, but wait until there is more experience with it” [24], and there is a fundamentally positive attitude among physicians [42].

Furthermore, concerning the current skepticism, hope can be placed in a generational change among the treating physicians as our data showed a correlation between the age of the participants and their attitude toward technology or their experience with technology. This can be explained by the fact that younger participants have already grown up with technology, which also fits with a Swiss study (“Among the very positively attuned physicians, a group with a high proportion of younger and stationary physicians could be identified” [42]) and other studies and articles worldwide [13,15,18,27,41]. Therefore, technology experience may become less important in the future because of the postmaturing generation of the physician workforce.

To close this gap, consideration should be given on the part of policy makers but also by developers to make the transition to digital methods easier and more attractive. For example, in other free-text fields in our study, physicians suggested offering a 30-day trial or providing better information and making the switch easier using explanatory videos, webinars, information sheets, or demonstration versions.

Nevertheless, a well-adjusted app has great potential to be used even in older groups of physicians because of saved effort and, therefore, also better payment (hypotheses 4 and 5). The time-saving argument was an important factor in our study, as in many others [12-19].

The answers to the exploratory questions also provide a picture consistent with the literature. For most physicians, an app must be declared as a medical device. This supports other surveys in which a binding test seal is demanded [39] and is one of the prerequisites for billability with the health insurance fund in Germany [34] and approval by the Food and Drug Administration in the United States [13], which in turn is considered important by most practitioners for the use of an app. Developers should focus on certification as a medical device, for example, because of the additional safety [35] or because of the health care system requiring it [13,34].

The question of whether physicians have concerns about data protection was predominantly answered with *rather applies*, which matches the results of other studies [18,22,24,25,27,32,33], indicates a great need for more education regarding the technology and its safety, and supports efforts such as a seal for consumers [40].

### Limitations

It becomes clear that developers of apps must pay attention to the relationship between the attitude toward technology and technology experience by the target group of physicians to make any age-appropriate adjustments that make it easier for physicians to use the app. Here, a less coarse division of the age groups would have been useful for a better assessment of the correlation between age and technology experience, which should be considered in further studies on this topic.

As general practitioners play an important role in dementia diagnoses because of their proximity to the patient [8], this study focused predominantly on them. However, these physicians are usually not specialists, and the results are not transferable to the entire medical profession. This has an impact on the final results as it can be assumed that some test persons are not as familiar with the clinical picture of dementia as specialists in the fields of neurology or geriatrics.

It would be beyond the limits of this work to survey physicians throughout Germany or even Europe or the world as a large proportion of participants were recruited via a circular mail from the regional Association of Statutory Health Insurance Physicians of the German federal state Baden-Württemberg. This results in a strong overrepresentation of participants from this federal state and, therefore, the results, assuming differences between east and west or north and south, cannot be generalized to Germany or beyond as a whole.

### Outlook

Research should be significantly expanded in the field of dementia screening app development to further reduce existing skepticism [18,22,24,25,27,32,33] and increase confidence in the technology. It should be noted that misdiagnosis using poorly developed apps is dangerous [8] and can have far-reaching consequences.

Some physicians also worry about an increase in time spent using apps to diagnose dementia in older people (*Secondary Research Questions*). As the samples in other studies on the DemPredict project were far too small to make a statement about the behavior of older people when using the app, we recommend further research in this field to positively encourage the medical profession to also rely on the technical assistance of the app for dementia diagnoses in older patients provided that it has been tested on older and already affected patients [46,53].

Further research is needed based on the study result that digital support is desired for early monitoring of the course of the disease in patients with dementia and the uniform picture that physicians can imagine having a digital test performed by a physician’s assistant (*Secondary Research Questions*). Here, elaboration and further interviews are necessary to create a clearer offer for the use of these 2 options.

Sentences such as “I don’t know if my clientele will engage with an app. They always look for a personal conversation with the doctor” (test participant 115) indicate a problem not considered in this study: acceptance (adherence) on the patient side, which of course also influences the use of the app by the physician. Here, future research should not lose sight of the connection and consider a meta-analysis from both sides of the likelihood of app use to create a more homogeneous picture of the factors influencing it. For this, interdisciplinary collaboration among technology, medicine, and psychology is essential, which is why we want to encourage further work in an interdisciplinary context.

Finally, the questionnaire developed in this study can also be used with minor adaptations in other medical fields that want to work with digital apps to obtain the opinions of medical specialists.

### Conclusions

The DHCQ revealed good test theoretical measures and showed signs of moderate to large correlations between the DHCQ dimensions *acceptance*, *attitude toward technology*, *technology experience*, *payment for the time of use*, and *effort of the collection* and the dependent variable *likelihood of use*. Although there were some critical voices within the group of physicians, it can be shown that there is a positive attitude and a disposition to cooperate in the development of supporting apps. It becomes clear that the likelihood of use of apps depends on more than a “good programmed app” but also requires interdisciplinary communication. The concerns about data protection and the fact that there are many apps on the market and few controls do not create an environment of confidence. However, if developers can gain the trust of physicians and mutual listening can take place to leverage the demonstrated correlations with age, experience, attitude, acceptance, effort, and payment, it is possible to work together to bridge these difficulties and enable better customization of apps to meet physicians’ needs.

Indeed, dementia is a disease that promises a better future if diagnosed early, but it is not the only one with a gradual progression and better prospects if diagnosed early. There is a great need for *good* and profitable measurement tools that are accepted by all stakeholders. However, this can only be achieved by involving physicians in the development of their working material.

## Appendix

Multimedia Appendix 1 The Digital Health Compliance Questionnaire developed and evaluated in this study.

Multimedia Appendix 2 Supplementary tables and figures with additional descriptive information.

## Abbreviations

CFA: confirmatory factor analysis
CFI: comparative fit index
DHCQ: Digital Health Compliance Questionnaire
DWLS: diagonally weighted least squares
TLI: Tucker-Lewis index
