# Supplementary material for: Digital Health Apps in the Context of Dementia: Questionnaire Study to Assess the Likelihood of Use Among Physicians
Source: JMIR Form Res. 2022 Jun 22;6(6):e35961. doi: 10.2196/35961 (PMC9260525; doi:10.2196/35961)
Supplement: Multimedia Appendix 1 [file formative_v6i6e35961_app1.docx]

**Multimedia Appendix 1. The Digital Health Compliance Questionnaire developed and evaluated in this study.**

**DF – Demographic Data**

**1. Age (item DF01)**

< 30

30-45

45-60

> 60

**2. Gender (DF02)**

male

female

diverse

**3. Specialization (DF03)**

General medicine (family doctor)

Specialist in neurology

Others: [free-text field] **(DF03_03)**

**4. Approval by statutory health insurance (Peculiarity of the German health care system) (DF04)**

Yes

No

**5. Do you diagnose dementia? (DF05)**

Yes

No

**6. If so, with which test? (DF06)**

Name of the test: [free-text field] **(DF06_01)**

**TE - Technology Experience**

**7. Do you have a smartphone or tablet? (TE01)**

Yes

No

**8. Do you know any applications that are supposed to make the work of doctors easier? (TE02)**

Yes

No

**9. If so, which ones? (TE09)**

name of the Application/s: [free-text field] **(TE09_01)**

**10. Are your patient files stored digitally or in paper form? (TE03)**

digital

paper form

mixed

**11. I am more confident than average when dealing with technology (TE04)**

1 - does not apply

2 - rather does not apply

3 - partly applies

4 - rather applies

5 - applies

**12. Technology has become an indispensable part of my everyday life (TE05)**

1 - does not apply

2 - rather does not apply

3 - partly applies

4 - rather applies

5 - applies

**13. I have always wanted to have the latest technical equipment (TE06)**

1 - does not apply

2 - rather does not apply

3 - partly applies

4 - rather applies

5 - applies

**14. I find it easy to learn to use an electronic device (TE07)**

1 - does not apply

2 - rather does not apply

3 - partly applies

4 - rather applies

5 - applies

**15. I am familiar with electronic equipment (TE08)**

1 - does not apply

2 - rather does not apply

3 - partly applies

4 - rather applies

5 - applies

**ET - Attitude towards technology**

**16. Technological progress has brought people mostly good things (ET01)**

1 - does not apply

2 - rather does not apply

3 - partly applies

4 - rather applies

5 - applies

**17. If we want to maintain our current standard of living, we have to keep pace with technological development (ET02)**

1 - does not apply

2 - rather does not apply

3 - partly applies

4 - rather applies

5 - applies

**18. I suspect that the work in my practice will change over the next few years as a result of technology (ET04)**

1 - does not apply

2 - rather does not apply

3 - partly applies

4 - rather applies

5 - applies

**19. Technological advances in the medical industry make me uncomfortable (ET05)**

1 - does not apply

2 - rather does not apply

3 - partly applies

4 - rather applies

5 - applies

**20. Electronic devices make my everyday life easier (ET09)**

1 - does not apply

2 - rather does not apply

3 - partly applies

4 - rather applies

5 - applies

**21. Electronic devices cause me stress (ET10)**

1 - does not apply

2 - rather does not apply

3 - partly applies

4 - rather applies

5 - applies

**22. Electronic devices make things much more cumbersome (ET11)**

1 - does not apply

2 - rather does not apply

3 - partly applies

4 - rather applies

5 - applies

**23. When dealing with technology, I am afraid of breaking something (ET12)**

1 - does not apply

2 - rather does not apply

3 - partly applies

4 - rather applies

5 - applies

**24. For me, technology is just a means to an end (ET13)**

1 - does not apply

2 - rather does not apply

3 - partly applies

4 - rather applies

5 - applies

**25. I am not afraid to try out technical devices (ET14)**

1 - does not apply

2 - rather does not apply

3 - partly applies

4 - rather applies

5 - applies

**IA - Information about the possible application**

**26. Potential dementia patients should be identified directly by their general practitioner through digital screening. The assessment of cognitive performance and thus the demarcation between healthy ones** **from people with mild cognitive impairments as well as dementia could be done by the patient by using this app independently, e.g. in the waiting room. This approach is intended to serve as a criterion for people in the outpatient environment and a low-threshold entry into the supply system - especially due to the enormous time savings on the part of the doctors.**

**As an example, the DemPredict is such an application and has already been tested for its usability in other studies (on a sample older patients) and their validity and reliability (on a sample of people with dementia). (IA02)**

In order to ensure the evaluability of the study, please describe briefly in your own words what the Application DemPredict should do.

Description: [free-text field] **(IA02_01)**

**EA - Application setting**

**27. A sensibly designed digital application can support an anamnesis just as well as a paper test (EA01)**

1 - does not apply

2 - rather does not apply

3 - partly applies

4 - rather applies

5 - applies

**28. I think that a digital application takes more effort than a paper test (EA02)**

1 - does not apply

2 - rather does not apply

3 - partly applies

4 - rather applies

5 - applies

**29. I fear privacy issues (EA03)**

1 - does not apply

2 - rather does not apply

3 - partly applies

4 - rather applies

5 - applies

**30. An automatic evaluation makes everyday practice easier for me (EA04)**

1 - does not apply

2 - rather does not apply

3 - partly applies

4 - rather applies

5 - applies

**31. I find it more time-consuming to use a digital application with older people than to use a paper test (EA05)**

1 - does not apply

2 - rather does not apply

3 - partly applies

4 - rather applies

5 - applies

**32. Networking the (anonymous) data collected on dementia would enrich research (EA06)**

1 - does not apply

2 - rather does not apply

3 - partly applies

4 - rather applies

5 - applies

**33. Networking the (anonymous) data collected on dementia carries the risk of misuse (EA07)**

1 - does not apply

2 - rather does not apply

3 - partly applies

4 - rather applies

5 - applies

**NW - Probability of use**

**34. I would prefer the application to the paper test if it saves me time (NW01)**

1 - does not apply

2 - rather does not apply

3 - partly applies

4 - rather applies

5 - applies

**35. I would offer dementia screening through an individual health service paid for by the patient himself/herself (in Germany called IGEL) in my practice (NW02)**

1 - does not apply

2 - rather does not apply

3 - partly applies

4 - rather applies

5 - applies

**36. I would request the following for such an service — dementia screening “via the application (NW08)**

Price in € (Euro): [free-text field] **(NW08_01)**

**37. I would prefer the application to the paper test if the screening is covered by the health insurance company (NW03)**

1 - does not apply

2 - rather does not apply

3 - partly applies

4 - rather applies

5 - applies

**38. I would prefer the application to the paper test if the results are directly available to me in the practice management system and I can save an evaluation by hand (NW04)**

1 - does not apply

2 - rather does not apply

3 - partly applies

4 - rather applies

5 - applies

**39. I would do more dementia screenings if the app shortened treatment time (NW05)**

1 - does not apply

2 - rather does not apply

3 - partly applies

4 - rather applies

5 - applies

**40. I would let the patient take the test in the waiting room without supervision (NW06)**

1 - does not apply

2 - rather does not apply

3 - partly applies

4 - rather applies

5 - applies

**41. I would have the patient work through the test under the supervision of an assistant (NW09)**

1 - does not apply

2 - rather does not apply

3 - partly applies

4 - rather applies

5 - applies

**42. If the application is certified as a medical product, this increases the likelihood of a use on my part (NW07)**

1 - does not apply

2 - rather does not apply

3 - partly applies

4 - rather applies

5 - applies

**43. According to the current state of knowledge, I would use the DemPredict application in my practice for dementia screenings (NW10)**

1 - does not apply

2 - rather does not apply

3 - partly applies

4 - rather applies

5 - applies

**44. I would use the DemPredict application for dementia screening under the following conditions (NW11)**

Requirements: [free-text field] **(NW11_01)**

**45. According to the latest scientific studies, dementia can be prevented or at least its course positively influenced by treating risk factors as early as possible (further information: Livingston et al., Dementia prevention, intervention, and care: 2020 report of the Lancet Commission. doi: 10.1016 / S0140-6736 (20) 30367-6).**

**As soon as there is a possibility to detect and monitor the risk factors of dementia in my patients at an early stage by means of digital support solutions, I would offer this support in my practice. (NW12)**

Yes

No

**WF - Further questions**

**46. ​​Do you need additional information from us in order to offer the app? (WF01)**

I would come to a presentation of the application to find out more  **(WF01_1)**

I would like a visit to my practice, where the application and its benefits are explained to me  **(WF01_02)**

I would ask my questions in a forum  **(WF01_03)**

Others: [free-text field] **(WF01_04a)**

**47. What would an alternative billing method be for you? (alternative to DiGA or IGEL as peculiarity of the German health care system) (WF02)**

Reply: [free-text field] **(WF02_01)**

**48. Which things are particularly important to you when using the application? (WF03)**

A sensitivity / specificity between 80% and 95% **(WF03_01)**

That the use of the application can be billed to the health insurance company **(WF03_02)**

That there is a recommendation from the Association of Statutory Health Insurance Physicians **(WF03_03)**

Others: [free-text field] **(WF03_04a)**

**49. Would you trust a test result if the patient performed the test beforehand at home? (WF04)**

Yes **(WF04_01)**

No **(WF04_02)**

Maybe **(WF04_03)**

**50. If you select "maybe" (WF07)**

Please explain your selection: [free-text field] **(WF07_01)**

**51. How exactly would you like the test results to be displayed? (WF08)**

I only need a diagnosis: Dementia, Mild Cognitive Impairment, Healthy **(WF08_01)**

I would like more detailed information in the form of a scoring **(WF08_02)**

I want to get a single view of the subtests **(WF08_03)**

Others: [free-text field] **(WF08_04a)**

**52. Additional remarks** **(WF05)**

If you have any further comments, criticism or concerns, please let us know! Only with your help

we can develop an application from which you as a medical profession can benefit.

Remarks: [free-text field] **(WF05_01)**
