# Supplementary material for: Digital Health Apps in the Context of Dementia: Questionnaire Study to Assess the Likelihood of Use Among Physicians
Source: JMIR Form Res. 2022 Jun 22;6(6):e35961. doi: 10.2196/35961 (PMC9260525; doi:10.2196/35961)
Supplement: Multimedia Appendix 2 [file formative_v6i6e35961_app2.docx]

**Multimedia Appendix 2. Supplementary tables and figures with additional descriptive information.**

| **item (*.r/*.m reversed pole)** | **dimension** | **mean** | sd | skew | **kurtosis** | se | **difficulty** | **discrimination** |
| --- | --- | --- | --- | --- | --- | --- | --- | --- |
| TE01 |  | 1.07 | 0.26 | 3.36 | 9.33 | 0.01 |  |  |
| TE02 |  | 1.70 | 0.46 | -0.86 | -1.27 | 0.03 |  |  |
| TE03 |  | 1.57 | 0.89 | 0.93 | -1.07 | 0.05 |  |  |
| TE04 | experience | 3.41 | 1.03 | -0.06 | -0.62 | 0.06 | 60.13 | 0.76 |
| TE05 | experience | 4.39 | 0.79 | -1.21 | 1.08 | 0.05 | 84.8 | 0.51 |
| TE06 | experience | 2.98 | 1.14 | 0.11 | -0.75 | 0.07 | 49.58 | 0.69 |
| TE07 | experience | 3.73 | 0.96 | -0.40 | -0.48 | 0.06 | 68.27 | 0.79 |
| TE08 | experience | 3.39 | 0.99 | 0.01 | -0.66 | 0.06 | 59.72 | 0.78 |
| ET01 | attitude | 3.61 | 0.76 | 0.11 | -0.25 | 0.04 | 65.28 | 0.60 |
| ET02 | attitude | 4.01 | 0.79 | -0.62 | 0.39 | 0.05 | 75.17 | 0.49 |
| ET04 |  | 4.31 | 0.77 | -1.15 | 1.73 | 0.04 | 82.72 |  |
| ET05.r | attitude | 3.23 | 1.09 | -0.14 | -0.52 | 0.06 | 55.81 | 0.62 |
| ET09 | attitude | 3.90 | 0.87 | -0.42 | -0.24 | 0.05 | 72.59 | 0.58 |
| ET10.r | attitude | 3.36 | 1.02 | -0.27 | -0.37 | 0.06 | 58.97 | 0.62 |
| ET11.r | attitude | 3.59 | 0.89 | -0.39 | 0.28 | 0.05 | 64.78 | 0.51 |
| ET12.r | attitude | 4.26 | 0.86 | -1.29 | 1.68 | 0.05 | 81.4 | 0.34 |
| ET13.r | attitude | 2.17 | 1.10 | 0.88 | 0.12 | 0.06 | 29.15 | 0.34 |
| ET14 | attitude | 3.92 | 1.01 | -0.76 | -0.16 | 0.06 | 73.09 | 0.44 |
| EA01 | acceptance | 4.03 | 0.97 | -0.89 | 0.34 | 0.06 | 75.83 | 0.46 |
| EA02.r | acceptance | 3.66 | 1.01 | -0.61 | -0.18 | 0.06 | 66.52 | 0.64 |
| EA03.r | acceptance | 3.54 | 1.21 | -0.65 | -0.56 | 0.07 | 63.62 | 0.42 |
| EA04 | acceptance | 4.12 | 0.91 | -1.03 | 0.90 | 0.05 | 77.91 | 0.63 |
| EA05.r | acceptance | 2.97 | 1.14 | -0.06 | -0.80 | 0.07 | 49.34 | 0.59 |
| EA06 | acceptance | 4.06 | 0.85 | -0.98 | 1.24 | 0.05 | 76.5 | 0.38 |
| EA07.r | acceptance | 3.15 | 1.18 | -0.34 | -0.81 | 0.07 | 53.82 | 0.48 |
| NW01 | use | 4.08 | 1.06 | -1.20 | 0.85 | 0.06 | 76.91 | 0.57 |
| NW02 |  | 2.24 | 1.24 | 0.71 | -0.62 | 0.07 | 30.90 |  |
| NW03 | use | 3.87 | 1.24 | -1.02 | 0.01 | 0.07 | 71.84 | 0.63 |
| NW04 | use | 4.27 | 0.99 | -1.64 | 2.41 | 0.06 | 81.73 | 0.63 |
| NW04.m | effort | 1.73 | 0.99 | -1.64 | 2.41 | 0.06 | 81.73 | 0.41 |
| NW05 | use | 2.13 | 1.26 | -0.99 | -0.14 | 0.07 | 71.76 | 0.59 |
| NW05.m | effort | 3.87 | 1.26 | -0.99 | -0.14 | 0.07 | 71.76 | 0.27 |
| NW06 |  | 2.64 | 1.22 | 0.40 | 0.76 | 0.07 | 41.11 |  |
| NW09.m | effort | 2.48 | 1.17 | -0.67 | -0.36 | 0.07 | 63.04 | 0.08 |
| NW07 |  | 3.38 | 1.29 | -0.46 | -0.97 | 0.07 | 59.55 |  |
| NW10 | use | 3.38 | 1.11 | -0.56 | -0.34 | 0.06 | 59.55 | 0.57 |
| NW12 |  | 0.99 | 1.21 | -7.45 | 59.24 | 0.07 |  |  |
| WF01 |  | 1.21 | 0.46 | 2.11 | 3.73 | 0.03 |  |  |
| WF01_01 |  | 1.26 | 0.44 | 1.11 | -0.76 | 0.03 |  |  |
| WF01_02 |  | 1.33 | 0.47 | 0.72 | -1.48 | 0.03 |  |  |
| WF01_03 |  | 1.25 | 0.44 | 1.13 | -0.72 | 0.03 |  |  |
| WF01_04 |  | 1.37 | 0.48 | 0.53 | -1.73 | 0.03 |  |  |
| WF03 |  | 1.94 | 0.90 | 0.07 | -0.93 | 0.05 |  |  |
| WF03_01 |  | 1.72 | 0.45 | -0.96 | -1.08 | 0.03 |  |  |
| WF03_02 |  | 1.63 | 0.48 | -0.56 | -1.70 | 0.03 |  |  |
| WF03_03 |  | 1.42 | 0.49 | 0.33 | -1.90 | 0.03 |  |  |
| WF03_04 |  | 1.17 | 0.37 | 1.79 | 1.19 | 0.02 |  |  |
| WF04 |  | 1.01 | 0.11 | 8.46 | 69.78 | 0.01 |  |  |
| WF04_01 |  | 1.19 | 0.39 | 1.58 | 0.49 | 0.02 |  |  |
| WF04_02 |  | 1.49 | 0.50 | 0.06 | -2.00 | 0.03 |  |  |
| WF04_03 |  | 1.34 | 0.47 | 0.68 | -1.55 | 0.03 |  |  |
| WF08 |  | 1.50 | 0.60 | 0.88 | 0.23 | 0.03 |  |  |
| WF08_01 |  | 1.29 | 0.46 | 0.91 | -1.18 | 0.03 |  |  |
| WF08_02 |  | 1.64 | 0.48 | -0.57 | -1.68 | 0.03 |  |  |
| WF08_03 |  | 1.52 | 0.50 | -0.07 | -2.00 | 0.03 |  |  |
| WF08_04 |  | 1.05 | 0.21 | 4.29 | 16.42 | 0.01 |  |  |

**Table S1: Item Analysis**

| **scale** | **mean** | sd | **median** | **range** | skew |  | **kurtosis** | se |
| --- | --- | --- | --- | --- | --- | --- | --- | --- |
| Likelihood of Use | 3.89 | 0.85 | 4.00 | 4 | -0.93 |  | 0.72 | 0.05 |
| Acceptance | 3.65 | 0.69 | 3.71 | 3.75 | -0.43 |  | 0.15 | 0.04 |
| Attitude | 3.56 | 0.59 | 3.56 | 3.11 | 0.06 |  | -0.15 | 0.03 |
| Experience | 3.58 | 0.80 | 3.60 | 3.20 | -0.02 |  | -0.70 | 0.05 |
| Effort | 2.11 | 0.77 | 2.00 | 4.00 | 0.81 |  | 0.72 | 0.04 |
| Payment | 3.87 | 1.24 | 4.00 | 4.00 | -1.02 |  | 0.01 | 0.07 |

Table S2: Scale Analysis

| **characteristics** | **number** | **%** |
| --- | --- | --- |
| **age** |  |  |
| <30 | 0 | 0 |
| 30-45 | 56 | 18.6 |
| 45-60 | 171 | 56.81 |
| >60 | 74 | 24.58 |
| **gender** |  |  |
| male | 138 | 45.85 |
| female | 161 | 53.49 |
| others | 2 | 0.66 |
| **specialization** |  |  |
| general medicine | 214 | 71.1 |
| neurology | 29 | 9.63 |
| internal medicine | 24 | 7.97 |
| psychology/ psychiatry | 31 | 10.3 |
| geriatrics | 1 | 0.33 |
| psychosomatic medicine | 2 | 0.66 |

Table S3: Socio-demographic Characteristics (n=301)

**

Figure S1: Acceptance and Likelihood of Use

**

Figure S2: Attitude and Likelihood of Use

**

Figure S3: Experience and Likelihood of Use

**

Figure S4: Payment and Likelihood of Use

**

Figure S5: Effort of collection and Likelihood of Use

Figure S6: Declared as Medical Device

**

Figure S7: Required Time for Older People

**

Figure S8: Digital Support for Early Monitoring

**

Figure S9: Use of the App by Patients under Supervision

**

Figure S10: Use of the App by Patients alone in the Waiting Room
